# Impact of Osteoporosis on Dental Implant Survival, Failure, and Marginal Bone Loss: A Systematic Review and Meta-Analysis

**DOI:** 10.3390/jcm14196719

**Published:** 2025-09-23

**Authors:** Su-Young Kim, Yoon-Jo Lee, Yei-Jin Kang, Seong-Gon Kim, Horatiu Rotaru

**Affiliations:** 1 Department of Oral and Maxillofacial Surgery, College of Dentistry, Gangneung-Wonju National University, 7, Jukheon-gil, Gangneung-si 25457, Republic of Korea; fmduddl20@gmail.com (S.-Y.K.); _yoonjo@gwnu.ac.kr (Y.-J.L.); 2 Department of Oral and Maxillofacial Surgery, Wonju Severance Christian Hospital, 20, Ilsan-ro, Wonju-si 26426, Republic of Korea; kyj292@hanmail.net; 3 Department of Oro-Maxillofacial Surgery and Implantology, ‘Iuliu Hatieganu’ University of Medicine and Pharmacy, 400012 Cluj-Napoca, Romania; dr.horatiu.rotar@gmail.com

**Keywords:** dental implants, osteoporosis, osteopenia, implant survival, implant failure, marginal bone loss, systematic review and meta-analysis

## Abstract

**Featured Application:**

This meta-analysis indicates that osteoporosis alone does not compromise dental implant outcomes, supporting safe and predictable implant therapy in older adults when guided by careful assessment and individualized planning.

**Abstract:**

**Background:** The influence of osteoporotic conditions on dental implant outcomes remains uncertain, with conflicting evidence regarding implant survival, failure, and marginal bone loss. **Methods:** A systematic review and meta-analysis were conducted in accordance with PRISMA guidelines (PROSPERO: CRD420251021400). PubMed and Scopus databases were searched for comparative human studies published between 2014 and 2024. Eligible studies compared implant outcomes in osteoporotic and non-osteoporotic patients. Risk ratios (RRs) and mean differences (MDs) with 95% confidence intervals (CIs) were calculated using fixed- or random-effects models. **Results:** Fourteen studies met the inclusion criteria, and seven provided sufficient data for quantitative synthesis. The pooled analysis demonstrated no significant difference in implant survival between patients with osteoporotic conditions and control groups (RR = 1.00, 95% CI: 0.97–1.02). Implant failure rates were also comparable (RR = 0.96, 95% CI: 0.59–1.56). Marginal bone loss showed no significant difference (MD = 0.22 mm, 95% CI: −1.69 to 2.12); however, substantial heterogeneity (I^2^ = 86%) was observed, requiring cautious interpretation. **Conclusions:** Osteoporotic conditions alone do not appear to negatively affect dental implant survival or failure. However, limited evidence and methodological variability highlight the need for prospective, standardized trials to confirm these findings and to clarify the role of antiresorptive therapy.

## 1. Introduction

Dental implant therapy has become a cornerstone for restoring oral functions and aesthetics in older adults, the fastest-growing demographic worldwide [1]. As this population expands, clinicians increasingly face systemic comorbidities that may influence peri-implant healing and long-term success [2]. Among these, osteoporosis—characterized by progressive loss of bone mass and micro-architectural deterioration—stands out for its high prevalence and potential to compromise osseointegration [3]. The intersection between implant dentistry and skeletal fragility has therefore become an important focus of contemporary research and clinical debate.

Osteoporosis affects more than 200 million people globally, with a disproportionate burden among postmenopausal women and the elderly [4]. Reduced bone mineral density (BMD) not only increases fracture risk in axial and appendicular sites but also alters the biomechanical environment of the jaws [5]. Although the mandible and maxilla benefit from unique embryologic origins and rich vascularity, their trabecular networks are still susceptible to systemic demineralization. Such changes may delay the cellular and molecular events necessary for osseointegration, thereby weakening primary implant stability—one of the strongest predictors of long-term survival [6].

Management of osteoporosis often involves antiresorptive agents such as bisphosphonates and denosumab, or anabolic drugs including teriparatide and romosozumab [7]. While these therapies reduce fracture risk, they also introduce implant-related concerns. Suppression of bone turnover could impair microscopic adaptation of peri-implant bone under functional load, and prolonged antiresorptive use has been linked to medication-related osteonecrosis of the jaws (MRONJ), a rare but severe complication in osteoporosis associated with invasive dental procedures [8]. Thus, balancing systemic skeletal benefits against localized oral risks poses a clinical dilemma for implant planning and execution in osteoporotic patients receiving antiresorptive therapy.

Despite extensive investigation, evidence remains inconsistent. Some studies suggest higher early failure rates or marginal bone loss in osteoporotic patients [9,10], whereas others report no significant differences compared with healthy controls [11,12,13]. Heterogeneity in study design, diagnostic criteria, drug regimens, follow-up duration, and implant systems has hindered the development of clear clinical guidelines. Consequently, decision-making often depends on anecdotal experience rather than robust evidence.

To address these uncertainties, this systematic review and meta-analysis evaluate the influence of osteoporosis on dental implant outcomes. We assess its effect on implant survival, failure, and marginal bone loss. By synthesizing available evidence, this review aims to establish an evidence-based framework to guide risk assessment, patient counseling, and surgical planning in older adults with osteoporosis.

## 2. Materials and Methods

This study constitutes a systematic review and meta-analysis. We formulated the research protocol following the guidelines provided by the Preferred Reporting Items for Systematic Reviews and Meta-Analysis Protocols (PRISMA-P) and registered it under the PROSPERO registration number CRD420251021400.

### 2.1. PICOS

The search methodology followed the PICO framework, representing P (Population), I (Intervention or Exposure for observational studies), C (Comparison), and O (Outcomes). In this systematic review, we applied the PICOS approach, encompassing the study population (patients clinically or radiographically diagnosed with osteopenia or osteoporosis), intervention (dental implant placement), comparison (implant placement in systemically healthy individuals without osteopenia or osteoporosis), and outcomes of interest (implant survival, implant failure, and marginal bone loss). The study designs included were prospective or retrospective cohort studies, non-randomized controlled trials, and case–control studies (Table 1).

### 2.2. Eligibility Criteria

Comparative studies (prospective or retrospective cohort studies, non-randomized controlled trials, and case–control studies) in humans assessing the implant survival rates, implant failure rate or implant marginal bone loss of people who are diagnosed with osteopenia or osteoporosis compared with healthy people without osteopenia or osteoporosis were included. The inclusion criteria for this study encompassed full-text articles involving dental implants, specifically those placed in patients who underwent antiresorptive medication treatment. The primary outcomes assessed were implant survival and implant failure. Eligible study designs included clinical trials, controlled trials, retrospective and prospective studies, and case series. To ensure the reliability and relevance of the findings, several exclusion criteria were applied. Studies were excluded if they lacked essential data on implant survival or implant failure, as incomplete data could compromise the comprehensiveness of the analysis. Articles published in languages other than English were not considered, and non-peer-reviewed literature—such as conference abstracts, posters, and unpublished studies—was excluded to maintain the quality and rigor of the evidence.

Research involving non-human subjects, including animal models, was excluded because their outcomes might not be directly translatable to human patients. Studies focusing on populations not representative of dental implant—such as individuals who underwent surgical fixation with plates and screws to treat craniofacial trauma—were also excluded. To include the most current evidence, studies published before a specified date were not considered. Duplicate studies or redundant data from the same study cohort were excluded to avoid repetition and ensure the integrity of the analysis. Lastly, articles lacking complete demographic information were not included, as such information is crucial for contextualizing the study results.

### 2.3. Information Sources

Following the guidelines outlined in the PRISMA statement, we conducted an electronic search of PubMed and Scopus. The manual search also included the bibliographies of all articles selected for full-text screening, as well as previously published reviews relevant to this systematic review. In our meta-analysis we included studies published between 2014 and 2024. Three reviewers (LYJ, KSY, and KYJ) performed study selection independently. In the event of disagreement between the reviewers, the fourth reviewer (KSG) was consulted.

### 2.4. Search Strategy and Article Selection

Searches of MEDLINE (PubMed) and Scopus were conducted. The search only included articles published in English, from year 2014 until 2024. We used the Boolean operators ‘OR’ to broaden the search and ‘AND’ to combine different areas. The search equations for each database were as follows:

- PubMed: (implant OR (dental implant)) AND (osteoporosis OR osteopenia OR (low bone density)) AND ((survive OR (survival rate) OR (success rate)) OR (failure OR fail OR (failure rate))).
- Scopus: (TITLE-ABS-KEY((“implant” OR “dental implant” OR “implant surgery”) AND (“osteoporosis” OR “osteopenia” OR “low bone density”)) AND ((“survive” OR “survival rate” OR “success rate”) OR (“failure” OR “fail” OR “failure rate”))) AND PUBYEAR > 2013 AND PUBYEAR < 2025 AND (LIMIT-TO (LANGUAGE, “English”)) AND (LIMIT-TO (SUBJAREA, “MEDI”) OR LIMIT-TO (SUBJAREA, “DENT”)) AND (LIMIT-TO (DOCTYPE, “ar”) OR LIMIT-TO (DOCTYPE, “cp”)).

Three independent reviewers, LYJ, KSY and KYJ, evaluated the titles and abstracts of all studies found in the initial search. If the abstracts did not provide sufficient information, the reviewers examined the full text to determine whether to include or exclude the studies. The authors reviewed the full texts of all remaining articles. Any discrepancies in the results between the reviewers were resolved by consensus, and if agreement could not be reached, a fourth researcher (KSG) was consulted.

**Figure 1:**
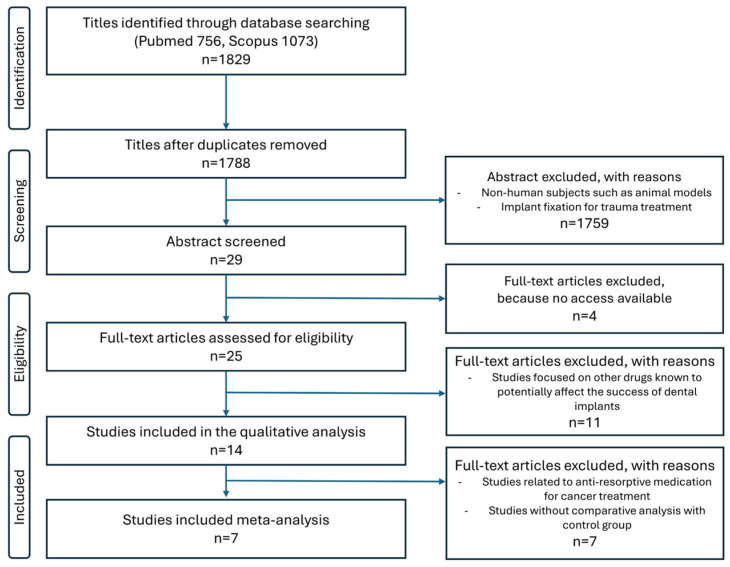
PRISMA flow diagram.

The PRISMA flow diagram provides an overview of the study selection process (Figure 1). Initially, the titles of the identified reports were screened, and duplicates were removed. Abstracts were reviewed if the titles suggested the study was relevant. For studies that appeared relevant or if the abstract was unavailable, a full-text analysis was conducted. Additionally, the references of identified papers and previously published systematic reviews on implant survival rates of patients who were treated with antiresorptive medication were cross-checked to ensure no articles were missed.

### 2.5. Data Collection Process

Data were extracted by two reviewers (LYJ and KSY) and entered into an Microsoft Excel 2019 spreadsheet (Microsoft, Redmond, WA, USA). The following items were collected: first author, year of publication, journal name, study design, number of patients, number of implants, mean patient age, types of experimental and control groups, types of antiresorptive agents and outcomes related to implant survival rate or peri-implant disease. Peri-implant disease was estimated by measuring bone level loss of implants during the follow-up period. To assess the level of agreement between the evaluators, the kappa statistic was employed using the same criteria as during the study selection phase. Any discrepancies were resolved through discussion between the evaluators; if consensus could not be reached, the assessor (KSG) was consulted for input.

### 2.6. Evaluation of the Study Risk of Bias

Due to the characteristics of the included studies—retrospective/prospective cohort studies, case–control studies, and other non-randomized designs—in this review, the RoBANS (Risk of Bias Assessment Tool for Non-randomized Studies) tool was used. There are five types of bias: selection bias, performance bias, detection bias, attrition bias, and reporting bias. The risk of bias was classified as either high, low, or uncertain. Risk of bias assessment was undertaken by two review authors (LYJ and KSY).

### 2.7. Statistical Analysis

For dichotomous outcomes, data were analyzed using RevMan 7.2.0 software, applying a fixed-effects model with the Mantel-Haenszel method to calculate the pooled risk ratios (RRs) with corresponding 95% confidence intervals (CIs). For continuous outcomes, the inverse variance method with a random-effects model was used to calculate the mean difference (MD) and 95% CIs. When data such as sample size, median, range, interquartile range, minimum and maximum values were available, the sample mean and standard deviation were estimated according to the established formula [14]. Heterogeneity among studies was assessed using Chi-squared (χ^2^) tests, degrees of freedom (df), the I^2^ statistic, and Tau^2^ values. To obtain more robust and reliable estimates, the Hartung-Knapp-Sidik-Jonkman (HKSJ) method was applied. Additionally, sensitivity analyses were performed by sequentially omitting individual studies to assess the robustness of the overall results and to evaluate the potential influence of any single study on the pooled effect estimates.

## 3. Results

The electronic search identified 1829 articles (PubMed: 756; Scopus: 1073). After removing 41 duplicates, 1788 titles and abstracts were screened. Of these, 1759 were excluded, primarily due to non-human studies or reports on implant fixation for trauma treatment. Twenty-nine abstracts were reviewed in detail, and 25 full-text articles were assessed for eligibility. Four studies were excluded because full text was unavailable, and seven were excluded because they investigated antiresorptive agents for cancer treatment or examined drugs other than those used for osteoporosis management that could confound implant outcomes. Ultimately, 14 studies met the inclusion criteria for qualitative synthesis, and 7 provided sufficient quantitative data for inclusion in the meta-analysis (Figure 1).

### 3.1. Study Characteristics

The 7 included studies [15,16,17,18,19,20,21] were published between 2015 and 2023 and comprised a mix of prospective clinical trials, retrospective cohort analyses, and case–control studies (Table 2). Sample sizes varied considerably, with patient numbers ranging from fewer than 20 to more than 700, and implant numbers from fewer than 50 to nearly 3000. Experimental groups consisted of patients with osteoporosis, diagnosed either by T-score thresholds or clinical assessment, with or without concurrent antiresorptive therapy. Control groups were systemically healthy individuals without osteoporosis.

The populations were predominantly older adults, often women over the age of 50, reflecting the demographic distribution of osteoporosis. Several studies reported a mean age above 60 years, and some explicitly restricted their inclusion to postmenopausal females. Patient sex distribution varied, but women were consistently overrepresented in the osteoporotic cohorts.

Outcome definitions were heterogeneous. The most common endpoints were implant survival and implant failure, with some studies also reporting implant success criteria, marginal bone loss, or patient satisfaction. Follow-up periods ranged from 1 to 7 years. While most studies relied on clinical and radiographic evaluations, a subset also documented supplementary parameters such as systemic medication use, smoking history, and comorbidities. Notably, some studies excluded patients with conditions such as diabetes, cardiovascular disease, or prior head and neck cancer to minimize confounding influences. Anti-osteoporotic medications varied across studies, with bisphosphonates (oral and intravenous) and zoledronic acid being most frequently reported.

**Figure 2:**
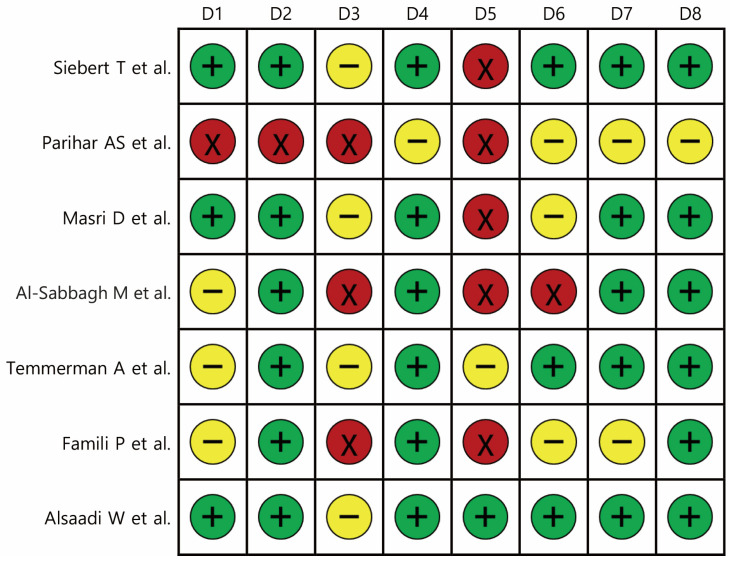
Risk of bias assessment for included studies across eight domains: D1, comparability of the target group; D2, target group selection; D3, confounders; D4, measurement of intervention/exposure; D5, blinding of assessors; D6, outcome assessment; D7, incomplete outcome data; and D8, selective outcome reporting. Green (+) indicates low risk of bias, yellow (–) indicates unclear or moderate risk, and red (×) indicates high risk [15,16,17,18,19,20,21].

Risk of bias was evaluated across eight domains (D1–D8) for all included studies. Overall, methodological quality was variable. Several studies (e.g., Siebert et al. [15] and Masri et al. [17]) demonstrated a predominantly low risk of bias, with consistent reporting and adequate methodological safeguards. In contrast, studies such as Parihar et al. [16] and Famili et al. [20] showed multiple high-risk domains, reflecting limitations in design and reporting transparency. Others (e.g., Al-Sabbagh et al. [18] and Temmerman et al. [19]) presented mixed profiles, with unclear or moderate risks in certain domains such as patient selection, outcome measurement, or follow-up adequacy. These findings underscore heterogeneity in study quality and should be considered when interpreting pooled results (Figure 2).

### 3.2. Implant Survival

**Figure 3:**
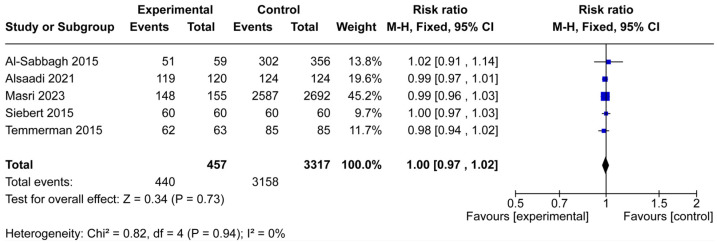
Forest plot of implant survival in osteoporotic versus non-osteoporotic patients. Five studies (457 osteoporotic, 3317 controls) were included. The pooled risk ratio showed no significant difference between the experimental and control groups (RR = 1.00, 95% CI: 0.97–1.02, *p* = 0.73), with no heterogeneity among studies (I^2^ = 0%). The blue squares represent the risk ratio of each individual study, with the size of the square proportional to the study’s weight in the meta-analysis [15,17,18,19,21].

Five studies comprising 3774 implants (457 in osteoporotic patients and 3317 in controls) were included in the meta-analysis of implant survival (Figure 3). Across individual studies, survival rates were uniformly high in both groups, with risk ratios ranging narrowly from 0.98 to 1.02. The pooled analysis showed no significant difference in implant survival between osteoporotic and non-osteoporotic patients (RR = 1.00, 95% CI: 0.97–1.02, *p* = 0.73).

Heterogeneity among the included studies was very low (Chi^2^ = 0.82, df = 4, *p* = 0.94; I^2^ = 0%), supporting the robustness of the findings. These results suggest that osteoporotic conditions, by themselves, do not adversely affect dental implant survival within the reported follow-up periods.

### 3.3. Implant Failure

**Figure 4:**
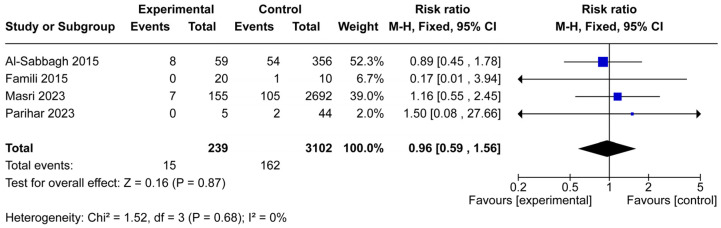
Forest plot of implant failure in osteoporotic versus non-osteoporotic patients. Four studies (239 osteoporotic, 3102 controls) were included. The pooled analysis revealed no significant difference in failure risk (RR = 0.96; 95% CI: 0.59–1.56; *p* = 0.87), with very low heterogeneity (I^2^ = 0%). The blue squares represent the risk ratio of each individual study, with the size of the square proportional to the study’s weight in the meta-analysis [16,17,18,20].

Four studies including 3341 patients (239 in the osteoporotic group and 3102 controls) reported data on implant failure (Figure 4). Overall failure events were rare, with only 15 failures observed in the experimental group and 162 in controls. The pooled analysis showed no significant difference in implant failure risk between osteoporotic and non-osteoporotic patients (RR = 0.96, 95% CI: 0.59–1.56, *p* = 0.87).

Between-study heterogeneity was negligible (Chi^2^ = 1.52, *df* = 3, *p* = 0.68; I^2^ = 0%), suggesting consistency across studies. Although individual studies varied in event frequency and sample size, the overall evidence indicates that osteoporotic conditions do not significantly increase the likelihood of implant failure.

### 3.4. Marginal Bone Loss

**Figure 5:**
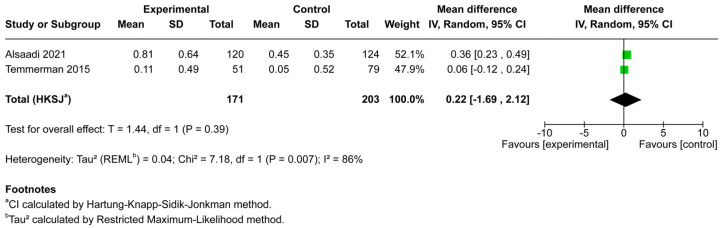
Forest plot of marginal bone loss in osteoporotic versus non-osteoporotic patients. Two studies (171 osteoporotic, 203 controls) were included. The pooled mean difference was not significant (MD = 0.22 mm; 95% CI: −1.69 to 2.12; *p* = 0.39). Substantial heterogeneity was observed (I^2^ = 86%), reflecting inconsistent findings across studies. The green squares represent the effect estimates for each included study, with the square size proportional to the study’s weight in the meta-analysis [19,21].

Two studies, including a total of 374 implants (171 in osteoporotic patients and 203 in controls), assessed marginal bone loss (Figure 5). The pooled analysis demonstrated no statistically significant difference in MBL between osteoporotic and non-osteoporotic groups (mean difference = 0.22 mm, 95% CI: −1.69 to 2.12, *p* = 0.39).

However, heterogeneity was substantial (Chi^2^ = 7.18, *df* = 1, *p* = 0.007; I^2^ = 86%), indicating considerable variability between the included studies. While one study suggested greater bone loss in the osteoporotic group [21], the other reported minimal differences [19]. Given the small number of studies and the high heterogeneity, the pooled evidence is insufficient to draw firm conclusions regarding the impact of osteoporotic conditions on peri-implant bone resorption.

## 4. Discussion

This systematic review and meta-analysis sought to clarify whether osteoporosis adversely affects dental implant outcomes. From 14 studies that met the inclusion criteria, seven provided sufficient data for quantitative synthesis (Figure 1). The pooled results demonstrated no significant difference in implant survival or implant failure rates between osteoporotic and non-osteoporotic patients (Figure 3 and Figure 4). Likewise, the limited data on marginal bone loss did not show a consistent association with osteoporosis (Figure 5). These findings suggest that osteoporotic conditions alone should not be regarded as a contraindication to implant therapy.

Our results are consistent with several earlier reviews and observational studies reporting comparable implant survival in osteoporotic and healthy patients. Yip et al. [9] similarly found no significant association between oral bisphosphonate use and implant failure among middle-aged women, while Cheng et al. [12] reported long-term implant survival of up to 20 years in women undergoing antiresorptive therapy. In contrast, some individual studies included in our analysis observed higher failure rates or greater marginal bone loss in osteoporotic patients, though these differences did not reach statistical significance when aggregated [19,21]. The discrepancy between single-study observations and pooled outcomes likely reflects differences in sample size, diagnostic criteria, and follow-up duration. Smaller studies may lack the power to detect subtle effects, while variations in osteoporosis severity and treatment regimens may influence outcomes heterogeneously.

From a biological perspective, osteoporosis is characterized by reduced bone mineral density and impaired trabecular architecture, both of which could theoretically impair osseointegration [22]. However, the jawbones differ from axial and appendicular bones in embryologic origin, vascularity, and remodeling dynamics, potentially mitigating systemic effects [23,24]. Moreover, the consistently high survival rates observed across studies suggest that once primary stability is achieved, peri-implant bone remodeling may be sufficient to maintain long-term osseointegration, even in osteoporotic bone [25]. Antiresorptive therapy adds further complexity. Bisphosphonates and denosumab are effective in reducing fracture risk but raise concerns about MRONJ [26,27,28]. In our analysis, few studies provided detailed stratification of implant outcomes by medication status, limiting our ability to disentangle the independent effects of osteoporosis and its treatments. Still, the absence of a clear detrimental effect of osteoporosis on implant outcomes may offer reassurance when planning implant therapy, provided patients are appropriately assessed and monitored.

Our risk of bias analysis highlighted considerable variability in methodological quality across included studies (Figure 2). While some prospective trials demonstrated robust design, adequate sample sizes, and consistent reporting, several retrospective cohorts and case–control studies exhibited methodological weaknesses, including incomplete follow-up, inadequate control for confounders, and unclear or inconsistent definitions of outcomes. This variability in study design and rigor substantially limits the strength of the pooled conclusions and reinforces the need to interpret the findings with caution. Importantly, the negligible heterogeneity observed in survival and failure analyses strengthens the reliability of these results, whereas the high heterogeneity in marginal bone loss outcomes reflects limited evidence and methodological inconsistency, underscoring the necessity for well-designed prospective studies with standardized protocols.

For clinicians, these findings suggest that osteoporosis alone should not be regarded as a contraindication to implant therapy. Instead, preoperative assessment should emphasize patient-specific risk factors such as systemic medication use, smoking, diabetes, and overall bone quality at the implant site, since these variables have been consistently linked to compromised implant outcomes [29,30,31]. Smoking impairs wound healing and increases the risk of peri-implantitis [31], while poorly controlled diabetes is associated with delayed osseointegration and greater marginal bone loss [30]. Furthermore, careful surgical planning, atraumatic techniques, and regular postoperative follow-up remain essential. Caution is warranted for patients receiving long-term antiresorptive therapy, as these individuals may face higher risks of impaired bone remodeling and MRONJ. Therefore, individualized risk assessment that integrates both systemic conditions and pharmacologic history should guide implant decision-making, rather than relying solely on osteoporotic diagnosis [27,28].

In addition to the intrinsic skeletal changes of osteoporosis, the pharmacologic management of the disease plays a pivotal role in implant outcomes [32]. Antiresorptive agents such as bisphosphonates and denosumab effectively reduce fracture risk but can suppress bone turnover and remodeling capacity, which are essential for peri-implant adaptation under functional load [33,34]. Long-term use of these agents has been strongly associated with MRONJ, particularly following invasive dental procedures [7]. Although the absolute risk of MRONJ in osteoporotic patients is lower than in oncologic populations receiving high-dose intravenous therapy, even this modest risk requires careful consideration [35]. The limited stratification of patients according to antiresorptive drug history in the included studies precludes a more definitive conclusion, but it is plausible that antiresorptive therapy may exert a greater influence on implant outcomes than osteoporosis itself. Consequently, clinical decision-making should not only account for the diagnosis of osteoporosis but also evaluate the type, dosage, and duration of concurrent pharmacotherapy.

Several limitations of this meta-analysis warrant consideration. First, the number of included studies was relatively small, especially for marginal bone loss, reducing the robustness of conclusions for this outcome. Second, heterogeneity in diagnostic criteria, implant systems, and follow-up intervals may have obscured subtle associations. Third, most included studies lacked randomized designs and were therefore subject to residual confounding. Importantly, stratification by antiresorptive therapy was inconsistent or incomplete, which limited our ability to distinguish the effects of osteoporosis itself from those of its pharmacologic management. Given the well-documented risks of impaired bone remodeling and MRONJ in patients on long-term antiresorptive therapy, this gap reduces the strength of our conclusions. In addition, the analysis of marginal bone loss exhibited substantial heterogeneity (I^2^ = 86%), likely reflecting differences in measurement protocols, outcome definitions, and follow-up durations across studies (Figure 5). This high variability reduces the certainty of pooled estimates and underscores the need for standardized peri-implant bone assessment methods in future research. Finally, stratification by type, dose, and duration of antiresorptive therapy was not consistently reported, precluding a more detailed analysis of pharmacologic influences. Future research should aim to address these gaps through well-designed prospective trials with standardized diagnostic criteria and outcome measures. Studies differentiating between untreated osteoporosis and pharmacologically managed cases, as well as long-term investigations into peri-implant bone remodeling, are needed. In addition, incorporation of patient-reported outcomes—including quality of life, satisfaction, and functional measures—would enhance the clinical relevance of future studies by capturing the patient perspective alongside clinical and radiographic outcomes.

## 5. Conclusions

This systematic review and meta-analysis, which included 14 studies qualitatively and 7 studies quantitatively, found no significant differences in implant survival, implant failure, or marginal bone loss between patients with osteoporotic conditions and systemically healthy controls. These results indicate that osteoporotic conditions alone may not compromise the predictability of dental implant therapy.

Nevertheless, the limited number of eligible studies, variability in diagnostic criteria, and inconsistent reporting of antiresorptive medication use restrict the strength of these conclusions. Additionally, some included studies combined osteopenic and osteoporotic patients, which may blur distinctions between these conditions and reduce the specificity of pooled findings. Future well-designed prospective trials, with standardized definitions of outcomes and stratification by osteoporosis severity and pharmacologic management, are needed to establish more definitive guidelines. Until then, careful preoperative assessment and individualized treatment planning remain essential when managing implant patients with osteoporotic conditions.

## Figures and Tables

**Table 1:** PICO framework used to define eligibility criteria for study selection.

Element	Description
P (Population)	Patients clinically or radiographically diagnosed with osteopenia or osteoporosis (hereafter collectively referred to as “osteoporotic conditions” unless otherwise specified)
I (Intervention/Exposure)	Dental implant placement
C (Comparison)	Dental implant placement in systemically healthy individuals without osteopenia or osteoporosis
O (Outcomes)	Implant survival, implant failure, marginal bone loss as reported by included studies
S (Study design)	Prospective or retrospective cohort studies, non-randomized controlled trials, and case–control studies
Focused question	Do patients with osteoporosis experience different implant survival, failure, or marginal bone loss outcomes compared with systemically healthy patients?

**Table 2:** Characteristics of included study.

Author (Year)	Study Design	Experimental Group	Control Group	Age/Sex	Patients			Implants		
					E	C	T	E	C	T
Siebert et al. (2015) [15]	Prospective clinical study	T-score < 2.5 with BP therapy	without osteoporotic disease based on t-scan	≥54/Females	12	12	24	60	60	120
Parihar et al. (2023) [16]	Retrospective study	diagnosed with osteoporosis	Healthy participants	35–65/(-) *	2	40	42	5	44	49
Masri et al. (2023) [17]	Retrospective cohort study	Diagnosis of osteoporosis A: Osteoporosis with BMT B: Osteoporosis without BMT	No osteoporosis	≤65: 459 implants, 66–79: 265 implants, ≥80: 68 implants/Male: 294 implants, Female: 498 implants	A: 46 B: 26	720	792	A: 155 B: 124	2692	2971
Al-Sabbagh et al. (2015) [18]	Retrospective study	Diagnosis of osteoporosis A: Osteoporosis with BMT B: Osteoporosis without BMT	No osteoporosis	59.4 ± 13.3/Male: 42% of patients, Female: 58% of patients	A: 39 B: 20	356	415	(-) *	(-) *	963
Temmerman et al. (2016) [19]	Prospective, non-randomized controlled study, multicenter	osteoporosis/osteopenia group subjects had a T-score ≤ −2	control group had a T-score of ≥−1	59–83(mean 69)	20	28	48	63	85	148
Famili et al. (2015) [20]	Case–control study	osteoporosis/osteopenia established by report of the treating medical physician	adequate bone density based on DXA measuring	52–70/Female	20	10	30	21	10	31
Alsaadi et al. (2021) [21]	Retrospective study	osteoporosis/osteopenia subjects with a T-score < −1	subjects with a T-score ≥ −1	60.62 ± 5.70/Female	27	26	52	120	124	244

* (-): no information.

## Data Availability

Data are contained within the article.

## Abbreviations

The following abbreviations are used in this manuscript:

BMD	Bone mineral density
MRONJ	Medication-related osteonecrosis of the jaws
PRISMA-P	Preferred Reporting Items for Systematic Reviews and Meta-Analysis Protocols
RRs	Risk ratios
CIs	Confidence intervals
MD	Mean difference

## References

[1] Aung Y.T., Eo M.Y., Sodnom-Ish B., Kim M.J., Kim S.M. (2024). Long-term survival rates of tapered self-tapping bone-level implants after immediate placement: A positional effective rationale. Maxillofac. Plast. Reconstr. Surg..

[2] Bérczy K., Göndöcs G., Komlós G., Shkolnik T., Szabó G., Németh Z. (2024). Outcomes of treatment with short dental implants compared with standard-length implants: A retrospective clinical study. Maxillofac. Plast. Reconstr. Surg..

[3] Cho J.M., Hong N., Rhee Y., Park W., Oh K.C., Seo Y., Lee H., Jo H.G., Shin Y., Kim J.Y. (2025). Clinical outcomes and bone marker changes in postmenopausal women with dental implants: A one-year prospective study. Int. J. Implant Dent..

[4] Srivastava M., Deal C. (2002). Osteoporosis in elderly: Prevention and treatment. Clin. Geriatr. Med..

[5] LeBoff M.S., Greenspan S.L., Insogna K.L., Lewiecki E.M., Saag K.G., Singer A.J., Siris E.S. (2022). The clinician’s guide to prevention and treatment of osteoporosis. Osteoporos. Int..

[6] Pieralli S., Spies B.C., Schweppe F., Preissner S., Nelson K., Heiland M., Nahles S. (2021). Retrospective long-term clinical evaluation of implant-prosthetic rehabilitations after head and neck cancer therapy. Clin. Oral Implants Res..

[7] Oh J.-H., Kim S.-G. (2024). Unveiling medication-related osteonecrosis of the jaw: A rapid review of etiology, drug holidays, and treatment strategies. Appl. Sci..

[8] Kim S.G. (2025). Nonessential amino acid is not nonessential in geriatric patients: Implications for maxillofacial wound healing and bone repair. Maxillofac. Plast. Reconstr. Surg..

[9] Yip J.K., Borrell L.N., Cho S.C., Francisco H., Tarnow D.P. (2012). Association between oral bisphosphonate use and dental implant failure among middle-aged women. J. Clin. Periodontol..

[10] Niedermaier R., Stelzle F., Riemann M., Bolz W., Schuh P., Wachtel H. (2017). Implant-supported immediately loaded fixed full-arch dentures: Evaluation of implant survival rates in a case cohort of up to 7 years. Clin. Implant Dent. Relat. Res..

[11] Tallarico M., Canullo L., Xhanari E., Meloni S.M. (2016). Dental implants treatment outcomes in patient under active therapy with alendronate: 3-year follow-up results of a multicenter prospective observational study. Clin. Oral Implants Res..

[12] Cheng Y.C., Ewers R., Morgan K., Hirayama M., Murcko L., Morgan J., Bergamo E.T.P., Bonfante E.A. (2022). Antiresorptive therapy and dental implant survival: An up to 20-year retrospective cohort study in women. Clin. Oral Investig..

[13] Grisa A., Veitz-Keenan A. (2018). Is osteoporosis a risk factor for implant survival or failure?. Evid. Based Dent..

[14] Kennel K.A., Drake M.T. (2009). Adverse effects of bisphosphonates: Implications for osteoporosis management. Mayo Clin. Proc..

[15] Siebert T., Jurkovic R., Statelova D., Strecha J. (2015). Immediate implant placement in a patient with osteoporosis undergoing bisphosphonate therapy: 1-year preliminary prospective study. J. Oral Implantol..

[16] Parihar A.S., Sonkar T.P., Mahabob N., Mohapatra A., Feroz S.M.A., Shetty M., Arasu T.U. (2023). Evaluation for survival of dental implants in medically compromised patients. Int. J. Chem. Biochem. Sci..

[17] Masri D., Masri-Iraqi H., Nissan J., Naishlos S., Ben-Zvi Y., Rosenfeld E., Avishai G., Chaushu L. (2023). On the association between dental implants, osteoporosis and bone modulating therapy. Appl. Sci..

[18] Al-Sabbagh M., Thomas M.V., Bhavsar I., De Leeuw R. (2015). Effect of bisphosphonate and age on implant failure as determined by patient-reported outcomes. J. Oral Implantol..

[19] Temmerman A., Rasmusson L., Kübler A., Thor A., Quirynen M. (2017). An open, prospective, non-randomized, controlled, multicentre study to evaluate the clinical outcome of implant treatment in women over 60 years of age with osteoporosis/osteopenia: 1-year results. Clin. Oral Implants Res..

[20] Famili P., Zavoral J.M. (2015). Low skeletal bone mineral density does not affect dental implants. J. Oral Implantol..

[21] Alsaadi W., AbouSulaiman A., AlSabbagh M.M. (2021). Retrospective study of dental implants survival rate in postmenopausal women with osteoporosis. Int. J. Dent. Oral Sci..

[22] Osterhoff G., Morgan E.F., Shefelbine S.J., Karim L., McNamara L.M., Augat P. (2016). Bone mechanical properties and changes with osteoporosis. Injury.

[23] Soares A.P., Fischer H., Aydin S., Steffen C., Schmidt-Bleek K., Rendenbach C. (2023). Uncovering the unique characteristics of the mandible to improve clinical approaches to mandibular regeneration. Front. Physiol..

[24] Omi M., Mishina Y. (2020). Role of osteoclasts in oral homeostasis and jawbone diseases. Oral Sci. Int..

[25] de Medeiros F., Kudo G.A.H., Leme B.G., Saraiva P.P., Verri F.R., Honório H.M., Pellizzer E.P., Santiago Junior J.F. (2018). Dental implants in patients with osteoporosis: A systematic review with meta-analysis. Int. J. Oral Maxillofac. Surg..

[26] Park C., Kim C., Park R.W., Jeon J.Y. (2024). Comparative effectiveness and safety outcomes between denosumab and bisphosphonate in South Korea. J. Bone Miner. Res..

[27] Khosla S., Burr D., Cauley J., Dempster D.W., Ebeling P.R., Felsenberg D., Gagel R.F., Gilsanz V., Guise T., Koka S. (2007). Bisphosphonate-associated osteonecrosis of the jaw: Report of a task force of the American Society for Bone and Mineral Research. J. Bone Miner. Res..

[28] Ruggiero S.L., Dodson T.B., Aghaloo T., Carlson E.R., Ward B.B., Kademani D. (2022). American Association of Oral and Maxillofacial Surgeons’ Position Paper on Medication-Related Osteonecrosis of the Jaws-2022 Update. J. Oral Maxillofac. Surg..

[29] Esposito M., Grusovin M.G., Coulthard P., Worthington H.V. (2006). The efficacy of various bone augmentation procedures for dental implants: A Cochrane systematic review of randomized controlled clinical trials. Int. J. Oral Maxillofac. Implants.

[30] Chrcanovic B.R., Albrektsson T., Wennerberg A. (2014). Diabetes and oral implant failure: A systematic review. J. Dent. Res..

[31] Levin L., Herzberg R., Dolev E., Schwartz-Arad D. (2004). Smoking and complications of onlay bone grafts and sinus lift operations. Int. J. Oral Maxillofac. Implants.

[32] Kwon Y.-D., Jo H., Kim J.-E., Ohe J.-Y. (2023). A clinical retrospective study of implant as a risk factor for medication-related osteonecrosis of the jaw: Surgery vs. loading. Maxillofac. Plast. Reconstr. Surg..

[33] Sulaiman N., Hanifah Y.Z., Mutalib N.-S., Cheung Y.-B., Duski I., Zainal H. (2023). Bisphosphonates and Dental Implants: A Systematic Review. Materials.

[34] Kim H.Y., Ha Y.C., Kim T.Y., Lee Y.K., Kim J.H. (2024). Long-Term Efficacy and Safety of Denosumab: Insights beyond 10 Years. Endocrinol. Metab..

[35] Kujanpää M., Vuollo V., Tiisanoja A., Laitala M.-L., Sándor G.K., Karki S. (2025). Incidence of medication-related osteonecrosis of the jaw and associated antiresorptive drugs in adult Finnish population. Sci. Rep..

